# Loosen control without losing control: Formalization and decentralization within commons‐based peer production

**DOI:** 10.1002/asi.24393

**Published:** 2020-06-16

**Authors:** David Rozas, Steven Huckle

**Affiliations:** ^1^ Faculty of Computer Science, Research Group on Agent‐Based, Social and Interdisciplinary Applications, Department of Computer Science Complutense University of Madrid Madrid Spain; ^2^ Centre for Research in Social Simulation, Department of Sociology University of Surrey Guildford UK; ^3^ Department of Informatics, School of Engineering and Informatics University of Sussex Brighton UK

## Abstract

This study considers commons‐based peer production (CBPP) by examining the organizational processes of the free/libre open‐source software community, Drupal. It does so by exploring the sociotechnical systems that have emerged around both Drupal's development and its face‐to‐face communitarian events. There has been criticism of the simplistic nature of previous research into free software; this study addresses this by linking studies of CBPP with a qualitative study of Drupal's organizational processes. It focuses on the evolution of organizational structures, identifying the intertwined dynamics of formalization and decentralization, resulting in coexisting sociotechnical systems that vary in their degrees of organicity.

## 1 INTRODUCTION

Drupal is a free/libre open‐source software (FLOSS) content management framework that provides a system for the development of websites. The Drupal project began in 1998, when Dries Buytaert and Hans Snijder developed a small content management framework for managing a message board at the University of Antwerp (Belgium); the system was released as FLOSS in 2001. It has grown ever since to become the backbone of 1.9% of websites worldwide.^1^ Furthermore, a community of more than 1.3 million people collaborate on Drupal.org.^2^

Over a 3‐year period of field‐based research of the Drupal community, this study furthers understanding of how a sizeable FLOSS community organizes itself by exploring both the development of source code and the organization of events. It focuses on the evolution of organizational structures (O'Mahony & Ferraro, 2007); it is framed not merely as a case of FLOSS, but as one of peer production common property (Schweik, 2009) and the broader phenomenon of commons‐based peer production (CBPP). Benkler (2002), who initially coined the term CBPP, refers to an emergent model of socioeconomic production in which groups of individuals cooperate to produce shared resources without a traditional hierarchical organization (Benkler, 2006).

In the Drupal community, we identify coexisting forms of organization resulting from two intertwined dynamics: the formalization of organizational processes and the decentralization of decision making. Drawing on the previous literature and collected data, this study investigates the emergence and evolution of Drupal's sociotechnical systems.

First, we examine work related to the organization of FLOSS and CBPP communities and provide an overview of the conceptual background of this study. We then introduce the research methodology used to study Drupal's organizational processes. Finally, we present and discuss our results.

## 2 CONCEPTUAL BACKGROUND

In an interview, Drupal's founder and lead developer Buytaert (2015) explained his views on how large FLOSS communities, such as Drupal, scale‐up decision making and continue to innovate:

[…] a Chief Digital Officer (CDO) of one of the largest mobile telecommunications companies in the world asked me how a large organisation […] should think about organising itself […] My advice to the CDO? Loosen control without losing control. […] the open source model grew around models of crowd‐sourced collaboration, constant and transparent communications, meritocracies, and a governance model that provides the platform and structure to keep the community pointed at a common goal.

Buytaert's idea of “loosen control without losing control” hints at the CBPP community's continuous struggle with formalization and coproduction processes (Arazy, Lifshitz‐Assaf, & Balila, 2019, p. 13). FLOSS studies and CBPP literature (e.g., Fuster‐Morell, 2010; Kostakis, Niaros, & Giotitsas, 2015; Zacchiroli, 2011) commonly describe governance^3^ as “do‐ocracies,” which consider a diverse set of interests and encourage open participation (Demil & Lecocq, 2006). Do‐ocracies feature peer‐review processes where decisions are decentralized, hence authority is assigned to individuals doing the work, who take responsibility for choosing roles and completing tasks. Such people gain community recognition and can influence organizational processes. The idea is that “no external body or hierarchy decides how actions should be carried out” (Fuster‐Morell, 2010, p. 282); therefore, do‐ocracies are in contrast to the more traditional “cathedral‐style” governance model (Raymond, 2001), which is characterized by top‐down forms of organization (Arazy et al., 2019, p. 13).

Benkler characterizes the governance of CBPP as controlled by any number of persons, under rules ranging from “anything goes” to “formal” (Benkler, 2006, p. 61). However, there has been criticism of such notions of do‐ocratic governance because they assume an all too simplistic model of egalitarianism; indeed, Mateos‐García and Steinmueller (2008) contend that FLOSS development comprises complex technological, social, and institutional elements. Kreiss et al. (2011, p. 247) argue that Benkler's work does not show how CBPP intersects with bureaucratic forms of organization. Furthermore, despite CBPP literature identifying decentralization as a critical concept (Arvidsson et al., 2017), there are very few empirical studies exploring how decentralization emerges—the rare exceptions are studies of Wikipedia, such as Viégas, Wattenberg, and McKeon (2007) and Forte, Larco, and Bruckman (2009). FLOSS studies tend to be limited because they explore the FLOSS phenomenon alone without widening their scope to a broader spectrum of CBPP (Gläser, 2007).

These critiques signal the need for a better understanding of the organizational processes of CBPP; in particular, the emergence and evolution of decentralized decision making. Indeed, there have been calls to understand the creation of effective forms of collective action and organization within CBPP (Txoler, 2014). This study responds to these; linking the study of organizational processes with activities in the collective production process (O'Mahony & Lakhani, 2011), thus contributing to research on both CBPP and FLOSS.

To this end, this study draws conceptually on activity theory (AT) (Engeström, 1987) and on Burns and Stalker's (1961) classic concepts of organic and mechanistic organizational structures.

**FIGURE 1:**
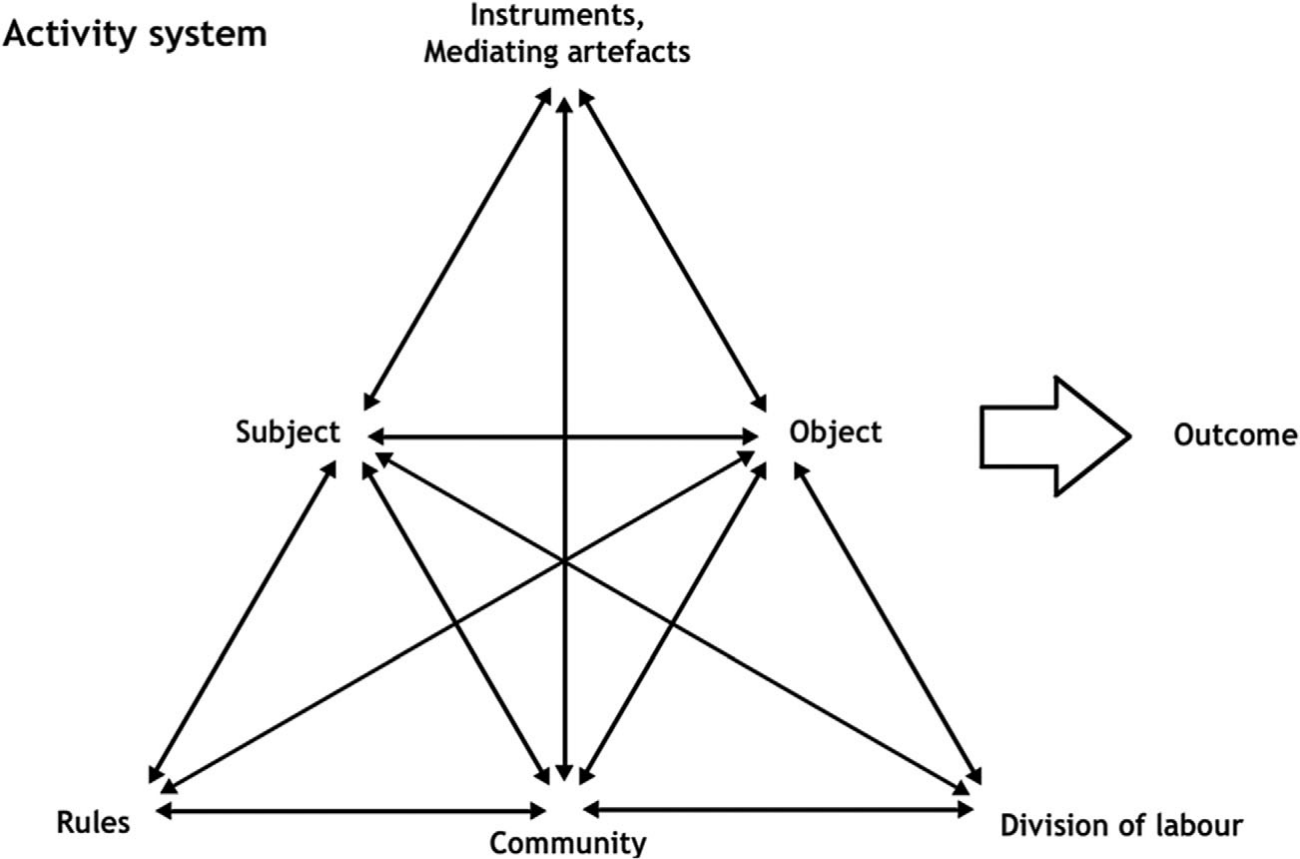
Engeström's activity system diagram. Retrieved from https://en.wikipedia.org/wiki/Activity_theory#/media/File:Activity_system.png under a CC‐BY‐3.0 license

We employ AT to establish cross‐contextual comparisons (Mason, 2002) from two types of collaborative activities with the highest degree of involvement and organizational complexity in Drupal: the development of source code and the organization of face‐to‐face (F2F) events. Throughout our application of AT, we explore the relationships between the different entities (see Figure 1) involved in these activities, providing the means to avoid simple monocausal explanations to explore the emergence of sociotechnical systems (Uden, Damiani, Gianini, & Ceravolo, 2007). Examples of cross‐contextual comparisons are those between the emergence of peer‐review practices to assess the quality of source code or the submission of a presentation to a community event, which emerged after exploring the relationships between the AT elements of these activities, such as rules, division of labor and the artifacts employed to collaborate.

The result of this analysis, sustained by AT, is the identification of a set of common organizational characteristics that result from the exploration of the sociotechnical systems of Drupal which have emerged around the development of code and the organization of F2F events. Because of these patterns, there was a need to ground the characteristics observed theoretically, leading us to draw on the organizational characteristics defined by Burns and Stalker (1961, pp. 119–122) for mechanistic and organic forms of organization. Higher degrees of formalization and centralization characterize mechanistic structures, where processes are bureaucratic and rigid, often involving high degrees of specialization, clear divisions of labor and where decision making follows a more top‐down hierarchy. In contrast, organic organizational structures have higher degrees of informality and decentralization, lacking rigid procedures and involving lower levels of specialization. If a division of labor is apparent at all, it is blurred, and specific needs drive the decision‐making processes. Next, we provide details of the methods, activities studied, as well as the application of these concepts in the analysis of Drupal.

## 3 MATERIALS AND METHODS

This section first details the study's methodological approach and the activities explored. Subsequently, it summarizes the data collection methods and analysis.

### 3.1 *Field‐based approach*

The first author collected and generated the materials employed in this article over 3 years of participant observation in the Drupal community—from 2013 to 2016.

When embarking on this research, the first author had been an active member of the Drupal community for over 3.5 years, therefore occupying the position of insider researcher (Brannick & Coghlan, 2007), whereby he had “natural access” (Alvesson, 2003) to the community. This previous exposure proved invaluable as it meant he understood the software and community. Thus, he avoided being considered a “newbie,” which eased access to certain field activities and facilitated a rapport with key informants. However, this prior experience also presented challenges related to the dynamics of insider research, such as role duality and preunderstanding (Brannick & Coghlan, 2007). Such issues were addressed by establishing a review process, rigorous introspection, and reflection on both the roles of researcher and Drupalista (a communitarian term to refer to members of the Drupal community).

### 3.2 *Activities studied*

The discussion below focuses on the different activities within Drupal which this study explores.

#### 3.2.1 *The development of source code*

**FIGURE A1:**
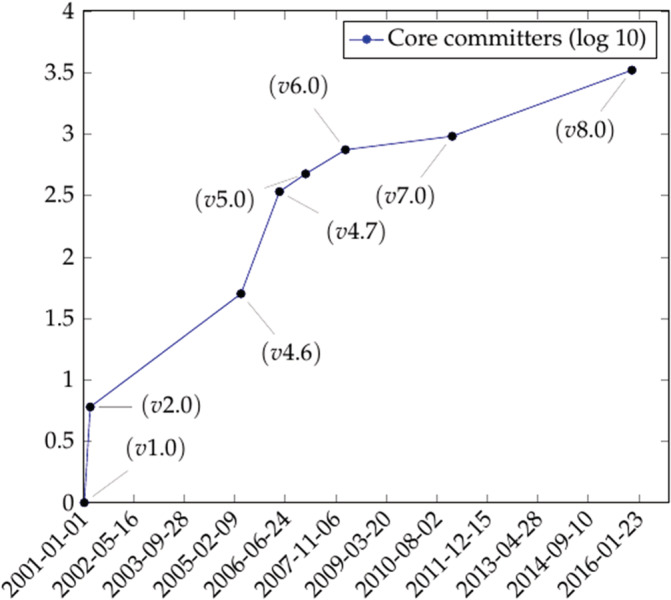
Number of core committers (log 10) per major Drupal release (Rozas, 2017). Based on the data collected by Zoubi (2016). The statistics from Drupal 3.0 to 4.5 could not be found, so they have been omitted. Retrieved from http://websolutions.hr/drupal‐history, under a CC BY‐SA 2.0 license [Color figure can be viewed at wileyonlinelibrary.com]

**FIGURE A2:**
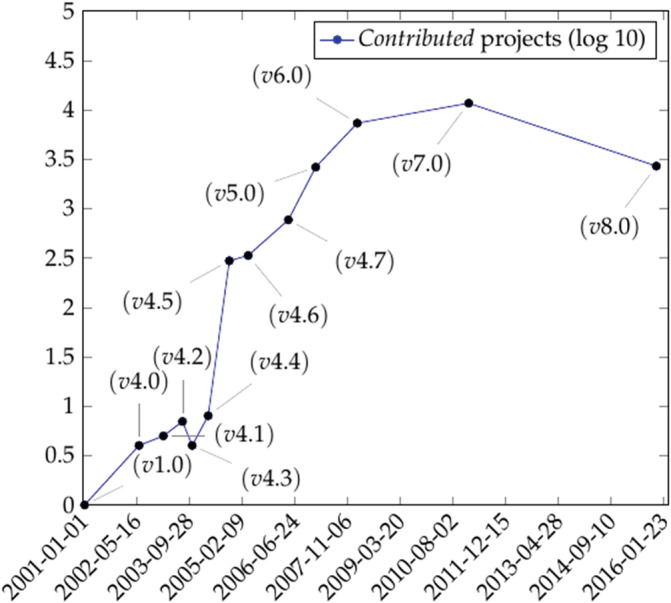
Number of contributed projects (log 10) per major Drupal release (Rozas, 2017). Based on the data collected by Zoubi (2016), drawing on captures from web.archive.org. The figure shows a constant growth of Drupal contributed projects. The exception to this reflects the time between Drupal 7 achieving stability and the release of Drupal 8, since contributed projects start development at the time the major release first appears. The statistics from Drupal 2.0 and 3.0 could not be found, so they have been omitted. Retrieved from http://websolutions.hr/drupal‐history, under a CC BY‐SA 2.0 license [Color figure can be viewed at wileyonlinelibrary.com]

For source code development, the analysis examined the dynamics of the development of three types of project: (a) core, (b) contributed, and (c) custom. A new installation of Drupal means an installation of its core, composed of a set of projects that provide basic functionalities. Thousands of participants maintain them (see Figure A1 in Appendix A). However, the power of Drupal resides in its extendibility. Typically, when looking to extend the core functionality of a Drupal installation, a developer will use a combination of contributed projects or build a custom project. There are more than 20,000 contributed projects from which to choose (see Figure A2 in Appendix A). Drupal.org provides the tools to share and improve such projects, which are maintained collectively by groups of Drupalistas. These projects form a rich set of digital commons that is vital to the success of Drupal since they meet a broad set of requirements. However, if core and contributed projects do not satisfy the required behavior, developers can create a custom project that does. Such projects are not always shared, but when they are, the Drupal community uses a quality assurance process to allow Drupalistas to apply for a custom project to become part of the sociotechnical system of contributed projects available on Drupal.org.

Table 1 provides a summary of the projects' characteristics explored.

**TABLE 1:** Main characteristics of Drupal projects

	Core projects	Contributed projects	Custom projects
Description	Official projects forming part of the default Drupal download	Official projects providing functionalities, not part of the core	Case‐specific projects created for a particular use
Transition	Drupal core may exclude a core project in a major release (regression)	Contributed projects can transition to become core projects	Custom projects can transition to become contributed projects
Platform availability	Drupal.org	Drupal.org and external platforms (e.g., github.com)	External, if any
Number available at Drupal.org	Tens	Thousands	N/A
Degree of formalization of peer reviewing	High	Medium	Low

#### 3.2.2 *The organization of F2F events*

For F2F events, the study involved three types of event: (a) DrupalCons, (b) DrupalCamps, and (c) local events.

The organization of F2F events has played a significant role in the day‐to‐day running of the Drupal community. The main focus of DrupalCons is peer‐reviewed presentations, community summits, code sprints, and social events. DrupalCamps are similar—they include peer‐reviewed presentations and code sprints; however, their scope is regional. Small‐scale local events, for example, “Drupal Beers” or “Drupal Show and Tell,” are informal meetups that typically present case‐studies or technical overviews. They are easier to organize and replicate. There are also local code sprints, hackathons, or “Drupal Coworking” events, where Drupalistas meet to work together on specific problems (Spinuzzi, 2012).

**FIGURE A3:**
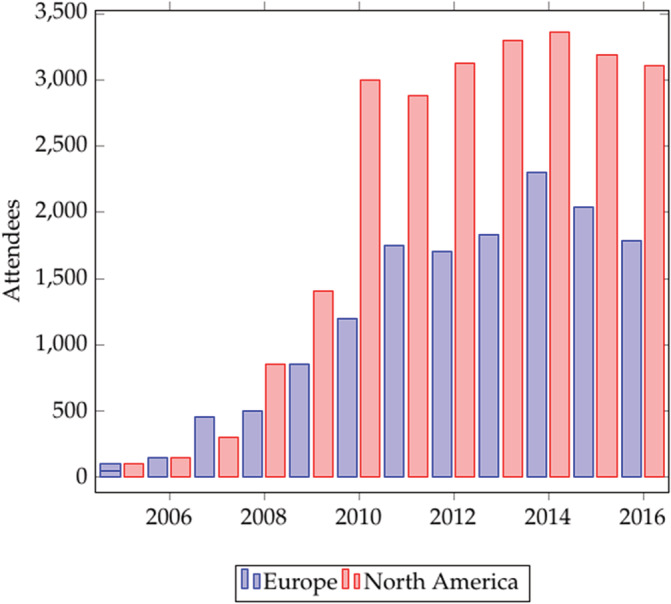
Number of attendees to DrupalCon events in Europe and North America (Rozas, 2017), based on the data reported by the Drupal Association (Drupal Association, n.d.‐a). See https://groups.drupal.org/events for further indicators on DrupalCamps and local events [Color figure can be viewed at wileyonlinelibrary.com]

In a similar way to the development of Drupal source code projects, F2F events have experienced significant growth (see Figure A3 in Appendix A) and different sociotechnical systems with significantly different characteristics have emerged over time. Table 2 provides a summary of the events studied.

**TABLE 2:** Main characteristics of Drupal events studied

	DrupalCons	DrupalCamps	Local events
Organizers	Drupal Association	Local groups, typically with help from other regional and national communities	Local groups
Scope	Global	Regional and national	Local
Frequency	Typically once a year in Europe and North America	Typically annually	Typically monthly
Total number of annual events	Two or three	Tens	Hundreds
Number of attendees	Thousands	Hundreds	Tens
Typical cost of a ticket	Hundreds of euros	Tens of euros	Usually free
Typical duration	1 week	2 or 3 days	Hours
Degree of formalization of peer reviewing	High	Medium	Low (if any)

### 3.3 *Data collection*

This article uses a qualitative approach combining 3 years of participant observation, analysis of an archive of 8,613 documents and 11 semi‐structured interviews.

#### 3.3.1 *Participant observation*

The first author began participant observation in October 2013, concluding in November 2016, including an analysis of both online and offline participation. He maintained field notes throughout.

Regarding online, he engaged in regular collaborative tasks, such as contributing to discussion groups, writing code, maintaining and coordinating official Drupal projects, creating documentation and conducting discussions via social networks and chat channels.

The first author's offline engagement was also varied, including F2F source code “sprints,” presentations at local events relating to this research, attending and participating in DrupalCamps and delivering a keynote presentation at a DrupalCon. In total, he participated in 32 F2F events (see Table B1 in Appendix B), the majority at local events in and around London, UK, as well as events in Madrid, Spain.

#### 3.3.2 *Documents collection*

Given the large nature of the Drupal community and the volume of information it generates, an initial point of collection of relevant documents was determined: Drupal Planet, which allows access to information beyond the official platform. The first author developed a set of scripts to automate the collection of links to 8,613 documents containing posts written by Drupalistas. The archive was monitored daily, inspecting new documents published. If they were relevant for the study, they were included for codification. Such documents were blog posts, presentations and discussions on Drupal.org about the organizational aspects of the community. Overall, the first author selected 875 significant documents. An overview of the data collected is presented at the end of this study in Table B2 (Appendix B).

#### 3.3.3 *Semi‐structured interviews*

In addition to hundreds of unstructured conversations carried out as part of the participant observation process, the first author conducted 11 semi‐structured qualitative interviews with participants involved in vital organizational processes (see Table B3 in Appendix B). This generated rich qualitative data, aiding a deeper understanding of Drupal's organizational processes.

#### 3.3.4 *Ethical considerations*

The first author followed the ethical principles described by the University of Surrey (Gallagher, 2013), as well as the recommendations from the Association of Internet Researchers (Markham & Buchanan, 2012). The use of data about any individual complies with the UK's Data Protection Act (Gov.uk, 1998). Individuals were anonymized in field notes and interviewees signed consent forms that allowed for the use of all materials gathered. Identity information included in the excerpts is the length of time a Drupalista has held an account at Drupal.org (up to the time of data collection) since this is a useful indicator of the member's interest in the community. Also included were gender and their roles within Drupal.

### 3.4 *Data analysis*

The first author used ethnographic content analysis (Altheide, 1987), involving a continuous process of discovery and comparison. The Computer‐Assisted Qualitative Data Analysis Software, Nvivo 10, was employed to support the analytical tasks. Despite its continuous nature, there were three distinctive data analysis phases.

During the first phase, the notion of “contribution activities” arose as the core category for classifying data. To understand all the contribution activities within Drupal, the first author participated in the development of Drupal projects and collaborated in the organization of F2F events, such as DrupalCamps. This informed interviewee selection, for example, since it allowed an understanding of the nature of their role.

Since contribution activities had become the central unit of observation for the study, the second phase incorporated AT as the primary analytical lens.^4^ AT facilitated the deconstruction of activities into several components in ways that allow cross‐contextual comparisons between them, even if, as in this case, the activities were substantially different. Thus, the elements of AT were incorporated as the main initial categories for each of the activities studied (see Tables 1 and 2) while collecting and coding the qualitative data during this second phase. Developing from these AT elements as categories, we explored the relationships between them. An example of the type of relationships explored is that between the artifacts employed for collaboration (e.g., the issues list of a contributed project in Drupal.org or the website to coordinate the organization of a DrupalCamp), the division of labor (e.g., Drupal roles such as being a maintainer of a contributed project or being a member of the peer‐reviewing team for presentations submitted at a DrupalCamp) played by participants and the implicit and explicit rules around such activities (e.g., coding standards for contributed projects, or codes of conduct for DrupalCamps). We then “followed the data” through such relationships to study how the changes led to, for example, the emergence of more formal peer‐reviewing processes to be defined over time to have a contributed project accepted officially in Drupal.org, or to evaluate presentations submitted to a DrupalCamp.

The third and final phase of data analysis involved employing the third generation of AT (Engeström, 2009) to conceptualize the growth experienced by Drupal and identify different sociotechnical systems that have emerged around these collaborative activities, not only as activities but as networks of them. For example, while the development of specific Drupal projects can be conceptualized as drawing on the aforementioned model of the second generation of AT (see Figure 1), the network of thousands of contributed modules in Drupal.org can be conceptualized as a sociotechnical system with distinct characteristics.

Examining public documents and discussions on Drupal.org enabled a trace of the history in the emergence of such systems, which subsequently led us to trace the identification of the general organizational dynamics of formalization and decentralization of decision making. The data related to the activity systems explored were grouped and analyzed according to the sociotechnical system to which they belonged. The result was the emergence of a set of categories describing organizational characteristics present in all the peer production activities explored (subsequently summarized in Tables 3 and 4). Examples of these are the degree of legitimation, to create the source code for an official project or to organize a communitarian event; the ease to participate in the contribution of code to such projects, or to give a talk at these events; or the degree of formality and quality assurance of the peer‐reviewing practices associated with them. These patterns led us to revisit the literature and, as a result, we employed the organizational characteristics of mechanistic and organic forms of organization, from the classic work of Burns and Stalker (1961), which helped us to group the organizational characteristics which emerged from our case study. We used Burns and Stalker's organic and mechanistic organizational forms as ideal types to carry out our analysis in the context of CBPP and, in line with other studies (e.g., Bahrami & Evans, 1987; Harrison & Rosenzweig, 1972; Louadi, 1998), we employed organicity as a continuum, therefore drawing on a *degree of organicity* (high, medium, and low) to present our findings.

**TABLE 3:** Summary of the degrees of organicity of the development of Drupal source code projects

Characteristics of organizational processes	High organicity	Medium organicity	Low organicity
Rules	**Only implicit rules**. Social norms on “writing good code”	**Formal rules partially affecting some areas**. Explicit coding style guides	**Rules affecting most areas**. Explicit mechanisms for quality assurance and division of labor
Specialization	**Blurred division of labor**. Commonly one developer, at times participants reporting bugs	**Some division of labor**. Maintainers of contributed projects as gatekeepers and committers	**High degrees of specialization**. Large number of specific roles, (product owners, core committers and release managers)
Formal processes	**Informal**. Social life (if any) organized around implicit social rules	**Some formality**. PAP for contributed projects	**Formal**. Bureaucratic processes for development of Drupal core
Coordination	**Uncoordinated action**. Permissionless culture	**Some degree of coordination**. Social norms for avoiding duplication of projects and promoting joining forces	**Coordinated**. Strongly coordinated, fear of a fork
Legitimacy	**Low level of legitimacy**. No expected legitimacy to create a custom project	**Intermediate necessary legitimacy**. To manage a contributed project	**High degree of legitimacy necessary**. To manage a core project
Perceived value of contribution activities	**Less valued**. Publishing a project not within Drupal.org	**More valued**. Maintaining a contributed project in Drupal.org	**Highly valued**. Maintaining a core project in Drupal.org
Ease of participation	**Easy to participate, organize or contribute**. Publishing a project as FLOSS out of Drupal.org	**Moderately easy to participate**. Publishing a contributed project in Drupal.org	**Difficult to participate**. Running a Drupal core initiative
Quality assurance	**No communitarian quality assurance mechanisms**. Projects not within the main collaboration platform are not attained to quality assurance	**Some level of quality assurance with specific mechanisms**. Explicit processes, such as the PAP for contributed projects	**High levels of quality assurance**. Mechanisms not only for projects but for decisions about the mechanisms themselves (core gates)
Required consistency	**Extremely fragmented and no need for consistency**. Low‐level of interdependency between projects not within the main collaboration platform	**Some levels of consistency**. Interdependency between some contributed projects	**Consistent**. Strong interdependency between all core projects

Abbreviations: FLOSS, free/libre open source software; PAP, Project Application Process.

**TABLE 4:** Summary of the degrees of organicity of the organization of events

Characteristics of the organizational processes	High organicity	Medium organicity	Low organicity
Rules	**Only implicit rules**. Social norms on “avoiding promotional talks” at local events	**Guiding rules**. Explicit selection criteria for DrupalCamp presentations	**Rules affecting most areas**. Explicit rules for how and who makes the rules, such as regulations regarding conflict of interest for DrupalCon Track Chairs
Specialization	**Blurred division of labor**. Roles allocated organically by participants in local events	**Some division of labor**. Explicit roles for organizers of DrupalCamps	**High degrees of specialization**. Explicit global and local roles for each track of DrupalCon sessions, to solve conflicts, etc.
Formal processes	**Informal and not requiring organizational structures**. No need for formal structures in local events	**Some formality**.The emergence of some local institutions, sometimes with legal forms, such as the Spanish and Moldovan Drupal Associations	**Formal**. Emergence of a formal, legal and global institution: Drupal Association
Coordination	**Uncoordinated action**. Permissionless culture	**Some degree of coordination**. Social norms on avoiding duplication of events nearby	**Coordinated**. Strongly coordinated, only organized by the global Drupal Association
Legitimacy	**Low level of legitimacy**. Anyone can do a “call” for a local event	**Some necessary legitimacy**. The organization of DrupalCamps through local institutions	**High degree of legitimacy necessary**. Organization of DrupalCons through contests of cities which present candidatures to the global Drupal association
Perceived value of contribution activities	**Less valued**. Speaking at a local event provides low symbolic capital	**More valued**. Speaking at a DrupalCamp provides intermediate symbolic capital	**Highly valued**. Speaking at a DrupalCon provides high symbolic capital
Ease of participation	**Easy to participate, organize or contribute**. Local events have low ratios of proposals, organizers sometimes have to persuade potential speakers	**Moderately easy to participate**. DrupalCamps have intermediate ratios of submissions/slots	**Difficult to participate**. DrupalCons have high ratios of submissions/slots
Quality assurance	**No communitarian quality assurance mechanisms**. Based on social norms	**Some level of quality assurance with specific mechanisms**. Selection of presentations by organizers at a DrupalCamp with specific criteria	**High levels of quality assurance**. Mechanisms not only for submissions but for decisions about mechanisms themselves, including conflict of interest regulation at DrupalCon
Required consistency	**Extremely fragmented and no need for consistency**. Low‐level interdependency between Local events	**Some levels of consistency**. Interdependency between DrupalCamps	**Consistent**. Strong interdependency between all DrupalCons

## 4 FORMALIZATION AND DECENTRALIZATION IN CBPP

This study has identified two primary organizational dynamics: formalization and decentralization of decision making.

Next, we present the characteristics of different sociotechnical systems which have emerged within Drupal around the activities mentioned above—developing source code and organizing events. We examine the degrees of formalization and decentralization within these systems and provide an overview of their interrelated organizational characteristics.

### 4.1 *High degrees of organicity*

In the Drupal community, sociotechnical systems with the highest degrees of organicity are the sociotechnical systems of local events and custom projects. Organizing a local event or publishing a custom project is reasonably straightforward. The primary organizational characteristics for these are their high degree of decentralization and ease of participation. An indicator of this organicity is their “permissionless” nature, a common characteristic in FLOSS development, reflected in local events. Indeed, Drupalistas such as I_1_ comment that, when compared with DrupalCamps and DrupalCons, such events have lower degrees of required legitimacy:

[…] I just saw some things [referring to other local events] were being organised […], and there was not any sort of Drupal Beers, which I'd seen was being organised in other cities. And I thought: “let's do one.” I didn't ask permission from anyone; I just did it. (Drupal developer and ex‐member of the Drupal Association Board of Directors, M, 8 years, originally in Spanish)

These events are organized by local communities and do not require the creation of more formal institutions for their sustainability. Instead, the main goal is for them to be easy to organize and replicate. The decentralized and fluid characteristics of this sociotechnical system can be compared with those related to the development of custom projects presenting the highest degree of organicity. There is no great need for coordination, nor fragmentation nor duplication. Instead, local events should be easily reproduced and spread. Following the source code metaphor, they can also be “forked”^5^ easily. The following excerpt about a discussion with a Drupalista illustrates this, referring to the organization of local events:

[…] after several issues with the organiser of the local events in his city, some Drupalistas decided to “fork” the local event: start a new type of meetup for people who didn't want to deal with the main organiser. […] I checked this out in meetup.org, and both groups are indeed co‐existing […]. (Field notes from an informal discussion with a Drupal developer at DrupalCon Amsterdam, October 1, 2014, M, 7 years)

Similarities can also be found with regard to decision making in these events, which is commonly implicit and based on direct participation, representing a fertile environment for the purest “do‐ocratic” forms of organization. The following excerpt by I_4_, while discussing how to become an organizer of a local event, illustrates this:

[…] There is no formal application process or whatever. It's basically, whoever shows up regularly, people get to know each other, and then, they work together. (Drupal core developer and mentoring organizer, F, 8 years)

Overall, these events and projects present a similar set of organizational characteristics, depicting what can be interpreted as sociotechnical systems of their own.

### 4.2 *Intermediate degrees of organicity*

An intermediate degree of organicity is evident in the emergence of numerous, distributed, and autonomous spaces in Drupal which require a higher degree of coordination and quality control than those above. Examples are DrupalCamps and contributed projects.

In the early years of Drupal, the transition of a custom project into the contributed sociotechnical system was based on an informal process of quality assurance. I_6_ explained how a single Drupalista was responsible for this process, as well as the informality and high degree of centralization at the time:

[…] at the time [around 2005–2006] the process was, you know, submit a tarball and *John* would review it, and if he liked it and it wasn't a duplicate, he would give you commit access to the one [EMPHASISING] CVS repository, that everyone was in. Which meant you had access to commit to every single project in CVS all at once. Oh, yeah… Wild West [LAUGHS]. (Drupal core developer and architect, M, 8 years)

This quote illustrates two key aspects present in this sociotechnical system during this early stage. Firstly, the action of gaining commit permissions and the perceived value this already had. This mechanism has been essential for decentralizing decision making related to the management of these digital commons while facilitating the scaling up of coordination. Secondly, this quote shows the existence of rudimentary monitoring mechanisms for projects to be incorporated into Drupal.org as contributed projects at the time. As in smaller FLOSS communities, they were based on social norms that regulate the expectation of the contributors to create good‐quality code as a way of generating trust. Projects that were not subject to these monitoring mechanisms, the aforementioned custom projects, had and have retained perceived low value for Drupalistas.

The perceived lack of value leads Drupalistas to favor the use of and contribution to contributed projects, hosted at Drupal.org, a more formalized sociotechnical system. However, contributed projects involve a lower degree of coordination compared to core projects. The following excerpt by I_6_ illustrates the differences between core and contributed sociotechnical systems:

[…] they are so different community‐wise. Drupal core is used by everybody in the community, right? A contributed module is only used by a fraction. Even if that fraction is high, even if 50% of the sites are using it, the hierarchies are a lot more flexible. They are basically a small group of maintainers, and they make all the decisions, and it's more… chaotic. […] So, it's just… contributed modules are like small Open Source projects. (Drupal core developer and architect, M, 11 years)

As part of the emergence and growth of the sociotechnical system of contributed projects, the Drupal community faced questions such as: who should be accountable for accepting and reviewing patches for a contributed project? How should it be decided whether a project is included or not? How is it possible to cope with the incremental need to review code?

Over time, the Drupal community formalized these processes in order to scale them up. From an AT perspective, Drupal's division of labor and the rules created within the system reflected this dynamic of formalization. A clear example of this is the definition of the Project Application Process (PAP): a quality assurance process carried out by the Drupal community which allows contributors to include a project as part of the sociotechnical system of contributed projects. Contributors must obtain a “git vetted role” that allows them to administrate the project, create new official releases and to extend the permissions to others, thus facilitating the decentralization of authority for decision making within the autonomous scope of that project. I_5_, a git administrator and key member in the organization of such processes, summarizes the workflow. He suggests that the contributed project becomes “real” when it receives a “nice URL” on the official platform:

[…] they open an issue on Drupal.org, where they explain the module and [provide a] link to the [sandbox].[…] Which is basically a git repository where you can push any code to. So the thing about those sandboxes is that they don't have a nice URL. They just have a number behind it. So they aren't that visible, and they don't have releases. […] [so when] they want to publish it as a real module under [a] certain namespace […] they go through the PAP. And then, basically, other community members review that code. (Drupal developer and git administrator, M, 8 years)

Similarly, DrupalCamps have their origin in BarCamps, which were more similar to local events presented in the previous section. While still organized by local communities, DrupalCamps have evolved to become full conferences. The following excerpt by I_6_, while reflecting on the changes in the organization of DrupalCamps over the years, illustrates this evolution:

DrupalCamps are […] community‐run. The [Drupal] Association for a long time did nothing for them, at all. They kind of grew up of BarCamps, so [in] the early ones […]. They evolved and, at this point, almost all of them are full‐on conferences with submitted sessions, and curation, and […] the same size as the PHP community conferences. 150 to 300 is typical. (Drupal core developer and architect, M, 11 years)

Two key aspects are illustrated here. Firstly, there was an increment in the organizational complexity when evolving to full conferences, transforming into a new sociotechnical system of events. These changes in the organizational processes entailed increased formalization. The second key aspect is the higher degree of decentralization and autonomy of the sociotechnical system overall when compared with that of DrupalCons.

This sociotechnical system has evolved following a trend of decentralization, facilitated by formalization. More formal structures have emerged, the level of organicity has reduced and, although more centralized than local events, the legitimacy of holding DrupalCamps remains within autonomous local communities, rather than the global institution, as in the case of DrupalCons. The following excerpt by I_2_ illustrates this distinction clearly:

If you look at DrupalCamps to DrupalCons, that's one of the big distinguishing factors. At a DrupalCamp, the Drupal Association might help. […] But they are in the background. […] The Drupal Association is for Drupal globally, […] at a national level, whatever it is: Drupal Association UK, Drupal Association Holland, whatever… It makes sense to run that locally. And I don't think that the Drupal Association has the infrastructure, time and capacity, let's say funds, to be involved […]. (Project manager, organizer of local events and DrupalCamps, M, 8 years)

The result was the emergence of a convoluted set of institutions created by local communities, as illustrated in the previous excerpt.

Overall, DrupalCamps feature similar dynamics of formalization to those of contributed projects. The organizational processes related to decision making for such events require a higher degree of community legitimacy. DrupalCamps demonstrate more explicit boundaries and rights to manage resources. More formal institutions emerge which facilitate how the individuals affected by the collective choices can participate in the creation of these rights. The result is a degree of flexibility because formalities are adapted to local circumstances.

### 4.3 *Low degrees of organicity*

The initial organizational processes of core projects were also informal. There were no explicit rules regarding decision making within projects, nor a clear division of labor or attributions. The following excerpt from I_6_ illustrates the informality of the organizational processes related to the development of core during the initial period:

[…] Once upon a time [he mentioned later “up to somewhere between Drupal 5 and 6” (2006–2008)] the process was: […] Dries opens up a new branch, and the committers are Dries and a few of his old friends. Pretty much just him and *Pete* […]. A new branch is open, people say: “Hey, I feel like working on X.” They go and work on X. (Drupal core developer and architect, M, 11 years)

This excerpt shows the relevance at the time, and perceived value of having the power to commit code or to create releases. Early on in Drupal core, Dries was responsible for creating releases, and he and his friend were the only Drupalistas with the power to commit to the core: acting as gatekeepers for quality control. These powers to commit and create releases are also relevant to understanding the decentralization of decision making concerning Drupal's organizational processes. However, with contributed projects it led to the emergence of a system in which the power was more distributed, with a higher degree of autonomy in the hands of project maintainers; in the case of core, the power to create releases and commit code remained more centralized.

Drupalistas explain this higher degree of centralization as addressing the need to coordinate a larger and constantly growing number of contributors. Changes in Drupal core affect the global direction of the project and, as a consequence, have also been subjected to higher degrees of quality assurance. This rigidity in the processes is also intertwined with a higher degree of legitimacy to carry out changes when compared with projects in the contributed system and, consequently, an even higher degree when compared with the custom projects system. The following quote by I_6_ illustrates the idea of how being able to perform large modifications in core used to operate on the basis of trust generated around the informal network explained in the previous quote, and how changes required, and continue to require, a higher degree of legitimacy and lack a “permissionless” nature—in the form of the approval of the project leader at the time:

[…] I was sitting on the floor, in the party room [during DrupalCon Sunnyvale (2007)] that night. […] And Dries comes by, sits down on the floor next to me. And… after I explained to him [the proposed changes] he said: “Yes, this makes sense, go with it.” Wait… what, what? [LAUGHS]. […] that kick in the pants to say: “OK, you have the project leader's blessing to do this big thing”. It was huge. […] a lot of people like to talk about Open Source: “You don't need permission to get involved,” but you kind of do when you are doing it at a high level when you are making large changes, you do need to have someone's blessing. […] And that blessing did help. (Drupal core developer and architect, M, 11 years)

Nevertheless, this does not imply that these processes were not affected by the aforementioned general dynamics of formalization and decentralization. A similar trend of decentralization in decision making could be observed over time as well, but this occurred in a more rigid environment when compared with that of contributed projects. The next quote, by I_4_, illustrates this increasing need to decentralize decision‐making processes and the relationship with greater formalization:

[…] it's so big and there's so many changes that there's no real way to do that informally. There needs to be a formal process so that the people who are responsible for doing certain things know that they're responsible for it, and know how to get it done. (Drupal core developer and mentoring organizer, F, 8 years)

I_4_ also explained how the trend toward increasing the degree of formalization reached a higher degree than in the case of the contributed system, which can be interpreted as these processes being subjected to the aforementioned higher degree of expected legitimacy as well as accountability to the community:

[…] procedures have to be more formalised in order for it to be welcoming for new contributors. Because people need to know how we do things, whom to talk to, and why. Otherwise, it looks like… […] you have to know certain people […]. (Drupal core developer and mentoring organizer, F, 8 years)

In the early stages the process strongly depended on the closeness of an informal network. As the community continued to grow, these processes incorporated more formalized mechanisms to improve transparency, objectivity and monitoring of peer‐production processes. An example of this formalization can be found during the transition from Drupal 7 to Drupal 8—5 years of development, a third of the life of Drupal—in the form of Core Gates. These are “checklists” of areas such as performance, accessibility and usability, which emerged in response to the need to define explicit quality assurance mechanisms that incorporate more Drupalistas' perspectives. Specific groups were created to participate in their elaboration, involving discussions by hundreds to thousands of participants, depending on the specific gate.^6^ Overall, these changes illustrate how the possibility to perform large modifications in these digital commons, or the power to change the direction of the project, became more distributed, from depending on an informal network towards depending more on explicit rules agreed and formalized by the community.

While this degree of decentralization is less than for the contributed sociotechnical system, a trend of decentralization can also be observed over time. Although, this results in a system characterized by a lower degree of autonomy, a higher degree of quality assurance in peer‐production processes, as well as a higher degree of legitimacy in order to manage these digital commons and perform changes.

Similar characteristics can be found in DrupalCons, which originated from the first international meeting in Belgium in 2005, considered by most Drupalistas as the first DrupalCon, as I_6_ explains:

[…] the first DrupalCon we had was actually in Antwerp, just because Dries was studying there […]. There weren't presentations. It was just kind of F2F discussions, people getting together to do some coding. (Developer and project manager, organizer of local events and DrupalCamps, M, 11 years)

**FIGURE 2:**
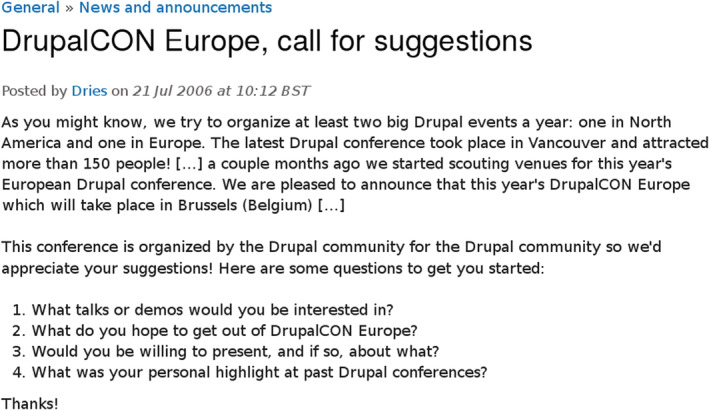
Excerpt from “DrupalCON Europe, call for suggestions.” Retrieved from https://www.drupal.org/node/74812 [Color figure can be viewed at wileyonlinelibrary.com]

This illustrates the high degree of organicity of these events at this early stage. The characteristics of these initial DrupalCons resemble those of current local events. The following excerpt, in Figure 2, depicts the thread^7^ opened by Dries asking for suggestions to organize the DrupalCon in Europe the year after, illustrating informality in the organization and the growth in attendance:

Some comments made in this thread by other Drupalistas illustrate the high degree of informality in decision making at the time: there were no formal rules or structures, the process was carried out following the purest forms of “do‐ocracy.” The consensus was reached via online discussion, in what was a small and informal network of people.

As the number of Drupalistas involved in the community grew, the organizational processes surrounding these events became more formalized. For instance, explicit rules for decision making and a clearer division of labor were defined for the selection of presentations for DrupalCons. This can be understood as part of the general dynamics of formalization and decentralization which shaped them, leading to the emergence of a sociotechnical system whose organizational characteristics more greatly resembled those of current DrupalCamps.

These changes should be understood in the context of the incipient foundation of the Drupal Association (2007). The emergence of an institution such as this can be understood as part of this general dynamic of formalization, as a consequence of the need to scale up organizational processes and decision making as the community grew; in a similar way to the emergence of local institutions for DrupalCamps, but with a global scope. As events continued to grow in attendance and organizational complexity, problems to scale up organization arose. In response, a transition towards a more precise definition of boundaries started, producing a shift in terms of jurisdiction, in which the Drupal Association explicitly retains the power to organize DrupalCons. I_6_, member of the board of directors of the Drupal Association at the time, explains his view on the reasons why this shift was necessary:

[…] We were burning out local teams. […] after [DrupalCon] Paris [2009], we made the decision to transition to the Association actually running these things. […] Chicago [2011] I'd say was the first modern DrupalCon, where the Association run it. There was a local team, but the Association owned the process […]. (Drupal core developer and architect, M, 11 years)

At first glance, this could be understood as a clear counter‐example of the general process of decentralization due to the reduction in the autonomy of local communities in the organization of DrupalCons. However, a more detailed inspection of the characteristics and outcomes of these events from a macro perspective indicates that this was a process of transition in which two different sociotechnical systems emerged. Similarly to the emergence of two different sociotechnical systems in the development of code (core and contributed), the higher degree of coordination and consistency of DrupalCons entailed the emergence of these new types of “modern DrupalCons,” whose role is different from earlier editions. The previous space was replaced by DrupalCamps, which represents a much more autonomous, organic and decentralized sociotechnical system. The following excerpt by I_7_ illustrates how DrupalCamps “filled in” this space:

[…] in Paris [2009] we were 600 people. Now it's what? 3,000 or something. […] because of size, it's maybe a different atmosphere. […] in Paris DrupalCon felt more like a DrupalCamp now. In terms of the closer community feel. […] DrupalCons have kind of lost something when they got bigger in terms of being a community event. But I think they gained something because they got the Camps to fit into that. (Project manager, organizer of local events and DrupalCamps, and volunteers' coordinator at several DrupalCons, M, 9 years)

Further examples are the emergence of a more explicit division of labor and rules for the processes surrounding the quality assurance and peer reviewing of presentations. For example, rules about the mechanisms for dealing with conflicts of interest for session chairs (Drupal Association, n.d.‐b), which in the case of DrupalCamps were unusual, and for the case of local events were based on social norms.

Overall, core projects and DrupalCons illustrate the emergence of Drupal's most mechanistic sociotechnical systems. The structures at this level require the highest levels of quality assurance and coordination, including greater consistency in the management of both core projects and events organization resulting in explicit rules, rather than social norms, as in the previous cases.

### 4.4 *Multiple organizational forms as a result of formalization and decentralization in CBPP*

Tables 3 and 4 summarize the characteristics of the organizational processes, according to their different degrees of organicity, that emerged from the analysis and have been explored in the previous sections. Table 3 shows the degrees of organicity of the organizational processes of the development of Drupal source code projects and Table 4 provides an overview of the degrees of organicity of the organization of events. These characteristics are not to be considered as isolated, but interrelated as part of a general organizational configuration related to the organization of CBPP activities in the Drupal community.

An example of the interrelationship between these organizational characteristics is that between perceived value, quality assurance and the degree of required consistency of the main object towards which, from an AT perspective, the action is directed: source code or an event. For instance, for the case of source code, the organizational processes of core projects require the highest degree of consistency. There are many dependencies in the object, the core, and a change in its code will affect all of the installations of Drupal websites worldwide. Hence, the addition of even a single line of code is subject to the strictest and careful quality assurance processes defined in the community. Having code committed to core projects is perceived as a highly valued contribution, and a sign of a greater reputation. On the contrary, code contributed to a project hosted outside of Drupal.org, for example in GitHub, does not require such consistency. Nor are there quality assurance processes regulated by the community, and these contributions are perceived as hardly valuable, if at all, and they are not reflected in the platform of collaboration—the artifacts from an AT perspective. For example, they are not acknowledged in the user profiles at Drupal.org. On the other hand, having code committed to a contributed project requires intermediate levels of quality assurance, which are enforced by the maintainers of that project. The perceived value depends on the complexity, popularity, and required degree of consistency of the project among other factors. For example, having a patch committed to a popular project in terms of installations, on which many other contributed projects and Drupal sites depend, requiring a higher degree of consistency, is perceived as a more valuable contribution than having code committed to a lesser known contributed project which is still in a sandbox status.

Another example of the interrelationship between these characteristics is that between the amount of explicit rules, division of labor, and quality assurance required for different degrees of organicity. For example, in the case of the organization of events and recalling the selection of presentations for DrupalCons, explicit rules are defined for the selection of the selectors with the explicit figures of “global and local track chairs” to ensure a high degree of quality assurance. In events with an intermediate degree of organicity, the selection might commonly be carried out by the whole group of organizers, who are informally self‐elected, and explicit rules for selectors do not commonly exist. However, there are explicit rules regarding the criteria for the selection of presentations, and, therefore, a high degree of quality assurance, although rules are not as exhaustive as for systems of the least organic degrees. For the most organic, however, there are no explicit rules for these selection criteria, and the process is wholly based on informal mechanisms of control (e.g., through social norms).

In other words, Tables 3 and 4 summarize how these multiple organizational forms, varying in their degree of organicity, interact and coexist transcending distinctions between online and offline mediums as well as the main focus of the activity itself: source code or events.

In sum, the growth experienced by Drupal has resulted in a general dynamic of decentralized decision making to distribute authority, which led to a general dynamic of formalization of organizational processes. The result has been the emergence of several sociotechnical systems which, while remaining oriented to similar activities (writing source code and organizing events), simultaneously interact and coexist. To manage these, Drupal has developed various organizational forms. The most formal systems ensure the successful execution of activities requiring the highest levels of coordination, consistency and quality assurance. Often this formalization is counterbalanced by decentralized, autonomous organizational forms that are fertile grounds for experimentation and innovation.

## 5 DISCUSSION

This study shows how Drupal's organizational processes have changed as the community has scaled up. Drupalistas state that they follow a “do‐ocratic” model (e.g., Byron, 2009; Fandy, 2012; Garfield, 2014), including several practices related to the idea of “bazaar governance” from FLOSS studies (Demil & Lecocq, 2006). That is, Drupalistas “work on what they want to work on, instead of being told what to work on” (Bacon, 2012, p. 514) and in which decision making is “made through consensus building and based on technical merit, trust and respect” (Bacon, 2012, p. 514). This “do‐ocratic” model implies that blurred and informal decision making become a source of tension when the organization scales up. However, the result was more formal and bureaucratic processes, due to a “lack of clarity around decision making […], inefficiency, and various community volunteer frustrations” (Buytaert, 2013).

Consequently, the picture that emerges of Drupal's modern‐day organizational processes is characterized by constant negotiation to distribute authority resulting in the emergence of several sociotechnical systems shaped by intertwined dynamics of formalization and decentralization of decision making. Today, Drupal's organizational structures distribute authority to scale up decision making, so that participants in peer production processes in these common property regimes (Schweik, 2009) “have authority to make at least some of the rules related to the use of that particular resource” (Ostrom, 1999, p. 528).

The identification of intertwined dynamics of formalization and decentralization in Drupal is congruent with Schweik and English's (2013) study, which uncovered statistical evidence of a trend towards formalization when FLOSS projects grow. Our findings are also congruent with Barberio et al.'s (2018, p. 164) study of the FLOSS Apache project, in which they identify the emergence of more formal boundaries over time and how they “are not simply existent or nonexistent, but a matter of degree” and how formalization does not necessarily decrease participation and “may increase porosity through clear rules.” In this respect, this study contributes to the FLOSS literature by providing an in‐depth account of how the dynamics of formalization and decentralization of decision making are intertwined, explaining the emergence of these different sociotechnical systems, as well as how these sophisticated organizational processes work in practice.

Regarding the CBPP literature, this study is in line with Viégas et al. (2007), who questioned the oversimplified accounts of Wikipedia's community at the time. They show the emergence of formal processes for selecting featured articles as the community grew. The subsequent work of Forte et al. (2009) focuses on the emergence of organizational structures. Similar to our findings for the Drupal community, Forte et al. (2009, p. 71) conclude that the story of the organizational processes within Wikipedia is that of increasing decentralization as complexity grew, whereby the community formalized processes. This study substantiates those findings by providing an in‐depth account of how such formalization occurs in the case of Drupal, contributing to CBPP literature on the understanding of how CBPP communities' organizational processes scale up.

Hence, we argue that although the concept of “do‐ocracy” describes some of the characteristics of peer production at the early stages, in the context of modern‐day Drupal, “do‐ocracy” should be understood as shared community values, which have been relevant in shaping how current organizational processes have evolved. Drupal demonstrates how to adapt from a “do‐ocracy” to become a successful example of large‐scale CBPP. Thus, we link the study of regulatory processes of CBPP with the actions in collective production processes in FLOSS. Therefore this article goes beyond the FLOSS models of “bazaar or cathedral” (Linux Congress, 1997; Demil & Lecocq, 2006) and shows further evidence of how these multiple forms of coordination coexist, as in Shaikh and Henfridsson's (2017) study of the Linux Kernel. If we are to develop on such a metaphor, this study shows how Drupal hybridizes the organizational forms of “bazaars and cathedrals,” or reveals a continuum of degrees of organicity rather than a dichotomy between mechanistic and organic organizational forms.

Finally, this case study provides a series of implications which might be useful for practitioners of FLOSS as well as wider forms of CBPP. These implications should be better interpreted as lessons learnt from the study of a large and global case of CBPP and FLOSS community, of interest to other CBPP communities coping with organizational challenges derived from growing in size and complexity, but which require to be adapted to their specific contexts. This is not an uncommon aspect in CBPP communities since, following a culture of exchange and learning from others' experiences, it is usual for CBPP communities to look at how others tackle certain issues to try to solve their own. For example, while working on their code of conduct, the Drupal community explored^8^ examples of other CBPP communities.

### 5.1 *Embracing varying organizational forms in peer production*

The simultaneous coexistence of several systems with different degrees of organicity focused on similar goals is sometimes perceived as inefficient and redundant by participants in CBPP communities. This coexistence is, however, useful to develop a certain equilibrium (e.g., formal/informal, global/local, or centralized/decentralized) between the dynamics of the community, which is well captured by the sentence “loosen without losing control” (Buytaert, 2015). CBPP communities should identify and embrace the coexistence of these various organizational forms in the community.

### 5.2 *CBPP institutions as umbrellas of initiatives*

CBPP institutions should assume organizational change as part of the day‐to‐day, rather than taking a position of resistance. A key aspect for them is to consider the need to create the conditions that enable the distribution of authority among several centers of governance that might emerge in the communitarian networks as CBPP communities grow. Rather than opting for imposing certain conditions from a position of central authority, their role should involve providing ways to coordinate the emergence and outcomes of these communitarian networks, for example in a federative manner, acting as an umbrella for communitarian initiatives.

### 5.3 *Offline matters*

Large and global CBPP communities, in which a significant amount of interactions are through online media, are commonly perceived as loosely connected (Benkler, 2006, p.60). This case study shows, however, a strong sense of community, which would not have been possible without the emergence of the sociotechnical systems around the organization of events. CBPP communities should consider the relevance of the offline medium to grow and sustain the health of the community. When growing in size and complexity, they should also try to envision ways to foster these interactions at several levels (e.g., both local and global) in order to scale‐up the sense of community.

### 5.4 *Limitations and future work*

While it was apt to select *activity* as the primary unit of observation and analysis for the study of a sizeable CBPP community, this choice also introduced limitations. For example, an observed general dynamic of professionalization emerged only tangentially from the data, as a distinction between paid and unpaid labor. This could be explored further by framing the Drupal community as a “community of companies performing projects,” rather than a “community of individuals undertaking activities” in which the main unit of analysis would be Drupal companies, as suggested by González‐Barahona, Izquierdo‐Cortázar, Maffulli, and Robles (2013). Other alternative approaches could consist of drawing on different concepts during the analytical phase, beyond the degree of organicity. These approaches could draw, for example, on several of Morgan's (1986, pp. 321–331) metaphors of machine, organism and brain to understand the complexity of the organizational life around Drupal.

Future research could also investigate interactions among sociotechnical systems. One such study could examine the influence exerted through mechanistic systems and the increasing degrees of formalization of more organic systems. For example, what is the impact of formalized testing practices from core projects on contributed projects? Alternatively, how are processes affected by the adaptation of the DrupalCon Code of Conduct in DrupalCamps? It may also be interesting to study influences in the reverse direction, too. For example, as arenas for experimentation and innovation, what is the role of contributed projects, and how do they influence the course of core projects?

## 6 CONCLUSIONS

This article studies the organizational processes of a large and complex CBPP community, Drupal, with an emphasis on activities surrounding the development of source code and the organization of F2F events. We explore the relationship between formal processes and the degree of decentralization of decision‐making processes. In sum, the growth experienced by the Drupal community has resulted in a general dynamic of decentralization, which encompassed the formalization of organizational processes.

As Drupalistas have established a constant process of negotiation to distribute authority, the result is the emergence of several sociotechnical systems which present different forms of organization varying in their degree of organicity. Systems oriented to similar activities, the development of source code and the organization of events, simultaneously interact and co‐exist. Overall, the most formal systems ensure the successful execution of activities requiring the highest levels of coordination, consistency and quality assurance. Often this formalization is counterbalanced by fully decentralized, autonomous organizational forms that are fitting for experimentation and innovation.

The identification of the intertwined dynamics of decentralization and formalization contribute to the literature by showing how the regulation of participation in FLOSS communities goes beyond the notions of bazaar governance and “do‐ocracies.” Instead, we show how different forms of organization in Drupal flourish and coexist.

## Endnotes

1 See “Usage statistics and market share of Drupal”—https://w3techs.com/technologies/details/cm-drupal/all/all.

2 Statistics self‐reported by the Drupal community at https://www.drupal.org/getting-involved. See Appendix A for growth indicators.

3 By governance, we draw on Fuster‐Morell's (2014) characterization as a complex system, which incorporates social norms and the roles played by the infrastructure.

4 See Rozas and Huckle (2020) for an in‐depth description of the application of AT for this case study, and the benefits, challenges and limitations for its application to more general forms of digital labor.

5 In FLOSS communities, a fork occurs when developers take a copy of source code from one project and start a new, independent, distinct version. This right is based on one of the main freedoms of FLOSS.

6 See https://www.drupal.org/core/gates and https://groups.drupal.org/node/166704 for further information about Core Gates. See also https://groups.drupal.org/node/158769 and https://groups.drupal.org/node/158759 for examples of discussions about Core Gates.

7 In these first events, there were no specific websites (e.g., https://events.drupal.org/barcelona2015) created for DrupalCons. The main artifacts for collaboration were pages at Drupal.org. For this specific event, see an announcement at https://www.drupal.org/node/74812 and a page in a Drupal group at https://groups.drupal.org/drupalcon-brussels-2006.

8 See https://groups.drupal.org/dcoc for information on, for example, discussions on how the Drupal Code of Conduct was influenced by those of other CBPP communities, such as Ubuntu or Wikipedia.

## Appendix A. INDICATORS OF THE GROWTH OF THE COMMUNITY

Below, we provide indicators of the aforementioned growth of the community for the activities studied. Figure A1 shows the number of source code committers (participants whose changes were officially accepted) whose work contributes to the development of Drupal core. Figure A2 shows the growth of peer‐reviewed contributed projects over time. Figure A3 presents the number of participants in DrupalCons in Europe and North America. The selection of these activities led us to explore the emergence of different forms of organization.

## Appendix B. DATA COLLECTED

Below, we present an overview of the data collected for this study. Table B1 shows participant observation at Drupal events. It includes the scope and the total number of events and days in which the first author participated.

**TABLE B1:** Summary of attendance at events

	Scope	Number of events attended	Total number of days
London Drupal beer and chat	Local	7	7
London Drupal show and tell	Local	8	8
Drupal Sprint weekend	Local	3	5
London Drupal coworking	Local	2	2
Drupal Madrid	Local	2	2
London Drupal learning	Local	1	1
London Drupal 8 release party	Local	1	1
Drupal Surrey	Local	1	1
DrupalCamp	National/regional/role‐specific	5	14
DrupalCon	International	2	12
		32	53

Table B2 shows an overview of the documents fully coded. The archive includes links to posts published in Drupal Planet (May 29, 2013 to November 23, 2016), yielding an archive of 8,613 links to documents. The archive continues automatically collecting links and is available at https://davidrozas.cc/lab/drupal_planet_archive.php. The source code is under a FLOSS license and available at https://github.com/drozas/drupal_planet_archive. Beyond the documents included for analysis as part of daily monitoring, several other materials considered relevant during participant observation were included for codification. For example, links to discussions in Drupal.org, documents mentioned during offline or online participant observation, user profiles, and digitized physical materials collected during participation in events.The grouping in Table B2 is by type of document and related sociotechnical system. The information includes the percentage of fully coded documents with respect to those automatically collected by the scripts.

**TABLE B2:** Summary of materials fully coded for documentary analysis

	DrupalCons	DrupalCamps	Local events	Core projects	Contributed projects	Custom projects	Total
Documents	158	85	31	368	58	15	715
Videos	3	4	1	17	2	0	27
Podcasts	6	5	1	18	0	0	30
Other datasets	14	12	10	45	17	5	103
Total	181	106	43	448	77	20	875
Percentage (per topic)	2.10%	1.23%	0.5%	5.20%	0.89%	0.23%	10.15%

Table B3 provides an overview of the interviewees' characteristics. Included are common general demographics, such as gender and nationality. Also displayed is Drupal‐specific information, such as the Drupalista's primary role and the number of years they had a user account on Drupal.org (up to the date of the interview). Also shown are the duration and mode of the interview.

**TABLE B3:** Overview of the interviewees' characteristics

Interviewee	Duration (min)	Mode	Gender	Nationality	Location	Account years	Main role
I_1_	195	In‐person	M	Spanish	London (UK)	8	Developer and themer
I_2_	69	In‐person	M	British	London (UK)	9	Project manager
I_3_	88	Video chat	M	Spanish	Stockholm (Sweden)	6	Developer and themer
I_4_	114	Video chat	F	USA	Chicago (USA)	8	Developer
I_5_	80	Audio chat	M	Austrian	Vienna (Austria)	8	Developer
I_6_	149	Video chat	M	USA	Chicago (USA)	11	Developer and system architect
I_7_	86	Video chat	M	British	Newcastle (UK)	9	Project manager
I_8_	51	Video chat	F	British	Brighton (UK)	4	Themer
I_9_	45	Video chat	M	Finnish	Helsinki (Finland)	6	Developer
I_10_	83	Video chat	M	Canadian	London (Canada)	6	Developer and themer
I_11_	117	Video chat	M	Danish	Copenhagen (Denmark)	11	Themer and project manager
